# The First Case of Severe Takotsubo Cardiomyopathy Associated with 5-Fluorouracil in a Patient with Abnormalities of Both Dihydropyrimidine Dehydrogenase (DPYD) and Thymidylate Synthase (TYMS) Genes

**DOI:** 10.7759/cureus.783

**Published:** 2016-09-14

**Authors:** Muhammad W Saif, Melissa Smith, Antonio Maloney

**Affiliations:** 1 Hematology/Oncology, Tufts Medical Center, Tufts University School of Medicine; 2 Oncology, Tufts Medical Center, Tufts University School of Medicine

**Keywords:** colon cancer, takotsubo cardiomyopathy, 5-fluorouracil, capecitabine, dihydropyrimidine dehydrogenase, thymidylate synthase

## Abstract

5-Fluorouracil (5-FU) is the backbone of the chemotherapy regimens approved for treatment of many malignancies, especially colorectal cancer (CRC). The incidence of cardiotoxicity associated with 5-FU ranges between 1.5% to 18% and is most commonly manifested as anginal symptoms. Cardiomyopathy is very rarely reported with 5-FU and capecitabine. A 35-year-old Caucasian male with T3, N1, M0 rectal cancer after the initial neoadjuvant chemoradiation with 5FU/LV followed by surgical abdominoperineal resection (APR), began mFOLFOX6 in the adjuvant setting. Following the first treatment, he developed severe cardiomyopathy, with a drop in ejection fraction (EF) to 19% from normal. The cardiac workup showed no ischemic or other etiologies to explain this cardiac event. He was a nonsmoker and only occasionally drank alcohol. He had no previous or family history of heart disease and had normal cholesterol level. He was treated for severe congestive heart failure (CHF). When the patient presented to us for second opinion, we decided to examine him for dihydropyrimidine dehydrogenase (DPD) deficiency and thymidylate synthase (TYMS) polymorphism. The patient was found to be heterozygous for the c.85T>C mutation, resulting in reduced DPYD enzymatic activity and homozygous for TYMS 5’TSER genotype 2R/2R *f. Our group first identified and reported P453L (1358C>T) type DPYD germline mutation in a patient who developed 5-FU induced cardiotoxicity. In this paper, we describe the first case of cardiomyopathy related to DPD deficiency and homozygous polymorphism of TYMS in a patient with colon cancer following 5-FU containing regimen. Fluorouracil-related cardiomyopathy has to be anticipated and treated to prevent the serious consequence of cardiac dysfunction. The prospective testing for DPD deficiency in patients might prevent DPD-deficient patients from severe toxicity or even death, and therefore the development of a unified screening method is warranted.

## Introduction

The association of DPD deficiency with severe toxicity after the administration of 5-FU and capecitabine has been reported [1]. DPD is the rate enzyme in 5-FU catabolism, and its deficiency results in the accumulation of 5-FU, potentially leading to an increased risk for developing a severe 5-FU-associated toxicity. The incidence of DPD deficiency is reported to be 2.7% in patients with cancer but partial DPD deficiency is much more common in 12.3% of black women, 4.0% of black men, 3.5% of white women and 1.9% of white men, respectively [2]. The TYMS enzyme is the primary drug target of 5-FU, leading to inhibition of DNA synthesis and cell death [3]. Investigators have researched the role of thymidylate synthase (TS) as both a predictive marker to response to 5-FU as well as to its toxicity but its role is not as well defined as for DPD.

Cardiotoxicity is a rare but serious toxicity associated with 5-FU and is thought to result mainly from coronary vasospasm [4]. Myocardial ischemia, cardiac arrhythmias, cardiac arrest, and sudden death are the most common cardiac complications following 5-FU [4]. Cardiomyopathy is a very rare cardiac toxicity, mainly reported as takotsubo cardiomyopathy (TCM), specifically related to capecitabine [5-8].

We report in this manuscript the case of a young patient with CRC who developed severe non-ischemic cardiomyopathy following the treatment with mFOLFOX-6 regimen (oxaliplatin 85 mg/m^2^, folinic acid 400 mg/m^2^ intravenous (IV) on day 1, followed by 5-FU 400 mg/m^2^ IV bolus and then 5-FU 2,400 mg/m^2^ IV over 46 h, administered every two weeks) and was later diagnosed to have abnormalities of DPYD and TYMS genes. Informed consent was obtained from the patient for this study.

## Case presentation

A 35-year-old Caucasian male with stage IIIB rectal adenocarcinoma initially diagnosed in January 2013 secondary to rectal bleeding and abdominal pain, presented to us for a second opinion regarding the management of recurrent metastatic rectal adenocarcinoma. The patient had a family history of a brother who died in his 30s of gastric cancer in the setting of Bloom syndrome (BS); however, the patient himself did not fit the classic phenotype and was reportedly tested in utero via amniocentesis.

Preoperatively, he received 5-FU by continuous intravenous infusion at a dose of 225 mg/m^2^/day concurrent with pelvic radiation (median 54 Gy/28 fractions) followed by surgical abdominoperineal resection (APR) with a colostomy. The patient was then started on mFOLFOX-6 as an adjuvant chemotherapy. However, he was admitted the same night due to acute cardiac toxicity manifested as acute pulmonary edema thought to be secondary to non-ischemic cardiomyopathy. ​Transthoracic echocardiogram (TTE) was remarkable for severe diffuse hypokinesis of the left ventricle (LV) and an ejection fraction of 19%. Cardiac catheterization and coronary angiogram showed no obstructive coronary artery disease and confirmed left ventricular wall motion abnormality as seen on the echocardiogram.​ The patient had no prior cardiac history, family history of cardiac disease, smoking or other cardiac risk factors. He received the standard neurohormonal therapy for heart failure in the intensive care unit (ICU) including an angiotensin-converting enzyme (ACE) inhibitor and beta blocker and he improved clinically. The cardiac computed tomography (CT) was negative for the coronary disease. The cardiac magnetic resonance imaging (MRI) during hospitalization and at four-weeks follow-up revealed myocarditis with late gadolinium enhancement (LGE) in the anterolateral LV extending to the apex (not in a coronary distribution) and improvement in ejection fraction (EF) to 55% and 62% respectively. As 5-FU was considered the culprit for the severe drop in EF, FOLFOX was discontinued immediately and the patient was switched to IROX regimen (irinotecan and oxaliplatin) with an initially reduced dose and later escalated to a full dose as he tolerated without difficulty. Follow-up transthoracic echocardiogram (TTE) six months later showed complete recovery of the cardiac function with an EF of 63%.

Unfortunately, the patient’s disease continued to progress despite multiple rounds of chemotherapy and he presented to us for a second opinion. While we discussed further chemotherapy options, I recommended testing him for genetic polymorphism for the enzymes related to 5-FU metabolism due to his cardiac history following 5-FU. In addition, this issue seemed more concerning as his younger brother died during chemotherapy treatment for advanced gastric cancer containing 5-FU. No autopsy was done.

The patient was found to be heterozygous for the c.85T>C mutation, resulting in reduced DPYD enzymatic activity and potentially increased risk of 5-FU toxicity. The TYMS 5’TSER genotype showed the enhancer region (5’TSER) on both alleles (2R/2R) of the thymidylate synthase (TYMS) gene. This 2R/2R genotype predicts low TYMS expression. While controversy exists in the literature, overall this finding predicts improved survival of patients with colorectal adenocarcinoma receiving 5-FU chemotherapy, but also increases the risk for 5-FU toxicity. 

Though no tissue was available to test his brother’s DPD, it is possible that he had DPYD or TYMS abnormalities that led to his death during the first cycle of 5-FU-containing chemotherapy.

## Discussion

Cardiomyopathy is very rarely reported with 5-FU and capecitabine. The literature review shows only a few cases of TCM associated with capecitabine [5-8]. TCM (transient apical ballooning syndrome, apical ballooning cardiomyopathy, stress-induced cardiomyopathy, Gebrochenes-Herz-Syndrom, stress cardiomyopathy) is a type of non-ischemic cardiomyopathy believed to be triggered by emotional stress such as the death of a loved one, a break-up or constant anxiety, lending it the name of broken-heart syndrome, in which there is a sudden temporary weakening of the muscular portion of the heart [9]. The criteria for the clinical diagnosis of TCM consist of:

1. Typical LV contraction pattern: transient hypokinesia, akinesis or dyskinesia in the LV mid segments with or without apical involvement accompanied with hypercontraction in the basal segments; regional wall motion abnormality (RWMA) that extends beyonds a single coronary artery vascular distribution; stressful trigger is usually but not always present;

2. Absence of obstructive coronary artery disease (CAD) or angiographic evidence of acute plaque rupture;

3. Newly developed electrocardiography (ECG) abnormalities (ST segment elevation and/or T-wave inversion) or modest elevation in cardiac troponin;

4. The absence of recent head trauma, intracranial hemorrhage, pheochromocytoma, myocarditis, or hypertrophic cardiomyopathy.

A review of the previously reported case reports of TCM secondary to 5-FU and capecitabine showed that none of the previous cases were either reported or tested for genetic polymorphism of the enzymes related to the metabolism of 5-FU and capecitabine. Our patient was found to be heterozygous for the c.85T>C mutation, resulting in reduced DPYD enzymatic activity. In addition, the TYMS 5’TSER genotype showed 2R/2R. The concomitant presence of two pharmacogenetics abnormalities of TYMS and DPYD are unique to our patient and has never been reported in the medical literature to the best of our knowledge.

As previously reported by our group, [1-2] DPD deficiency is a familiar syndrome secondary to the allelic mutations in the DPYD gene. DPD deficient patients are prone to develop life-threatening toxicities from 5-FU therapy as seen in our patient and probably his brother. A direct association in 5-FU related cardiotoxicity and DPD deficiency has not been established except in the anecdotal reports.

TScatalyzes the intracellular conversion of deoxyuridylate to deoxythymidylate. 5-fluorodeoxyuridylate, the active metabolite of 5-FU, binds to TS and inhibits TS by forming a stable ternary complex. The human TS gene (TYMS) is also considered a candid factor for 5-FU toxicity and efficacy. The TYMS gene is polymorphic, with either double (2R) or triple (3R) tandem repeats of a 28-base-pair sequence downstream of the cap site in the 5'-terminal regulatory region [3,10]. The presence of triple repeats predict not only intratumoral TYMS mRNA expression but also a response to 5-FU. Patients with the 2R/2R genotype had the most favorable response to 5-FU therapy because the intratumoral TYMS mRNA expression is significantly lower in this group. However, 2R/2R variation of TYMS is also associated with a ≤ 2.5-fold risk of toxicity to 5-FU/capecitabine. On the other hand, patients with the 3R/3R genotype had significantly less toxicity compared with the 2R/3R and 2R/2R genotype with 5-FU based chemotherapy. This may be due to the expression levels in the normal tissue. The increased TYMS mRNA expression in both the normal and tumor tissue of patients with the 3R/3R genotype protects the cells against damage by 5-FU treatment because of the low efficacy of TS inhibition. The resulting decreased cell death rate leads to a resistance (tumor tissue) and low toxicity (normal cells). The data suggests that the lower TYMS mRNA level in the normal tissue of patients with 2R/2R or 2R/3R genotype might enhance the cytotoxic effects of 5-FU, leading to more severe side effects in these patients.

This case demonstrates the increasing recognition of life-threatening adverse events, including one of the first reports of dilated cardiomyopathy (DCM) associated with 5-FU toxicity and genetic abnormalities responsible for the metabolism of the culprit chemotherapeutic agent. It establishes a correlation between severe 5-FU-associated cardiac toxicity and DPD deficiency, as well as association with TYMSabnormality. This case emphasizes the importance of early recognition of DPD* *deficiency to prevent adverse events of chemotherapy. Testing for DPD deficiency is currently not considered cost effective, and we lack a uniform screening method. Prospective testing for DPDdeficiency in patients might prevent DPD-deficient patients from severe toxicity or even death, and therefore the development of a unified screening method is warranted. The polymorphism of TYMS is of significance to the expression of TS, which might influence the biology and 5-FU sensitivity. Further prospective studies in a larger number of patients are also needed to confirm the association between 5-FU and TYMS genotypes that could be used in the allocation of patients for treatment approaches and for decisions on follow-up programs.

## Conclusions

Non-ischemic dilated cardiomyopathy in the setting of 5-FU therapy is a relatively uncommon phenomenon. Multiple factors underlying the pathogenesis have been postulated including direct myocardial damage, coronary vasospasm, platelet aggregation, endothelial damage, increased metabolism leading to energy depletion and ischemia, oxidative stress causing cellular damage, and autoimmune phenomena. It is prudent for physicians to be aware of the rare form of cardiotoxicity of 5-FU and its analogs, and they should consider genetic testing as well as a complete cardiac evaluation before re-challenging the patient. Fluorouracil-related cardiomyopathy has to be anticipated and treated to prevent the serious consequence of cardiac dysfunction. The issue of measurements of DPD activity and other pharmacogenetic markers may be useful in high-risk patients scheduled to receive fluoropyrimidine-based chemotherapy but not in routine practice.
